# Comparative risk of serious infection among biologic therapies for inflammatory bowel disease in pediatric patients: A target trial emulation

**DOI:** 10.1002/jpn3.70251

**Published:** 2025-11-25

**Authors:** Serena Yun‐Chen Tsai, Ashwin N. Ananthakrishnan, Harland S. Winter, Kevin Sheng‐Kai Ma

**Affiliations:** ^1^ Department of Dermatology Massachusetts General Hospital, Harvard Medical School Boston Massachusetts USA; ^2^ Division of Gastroenterology, Department of Medicine Massachusetts General Hospital, Harvard Medical School Boston Massachusetts USA; ^3^ Department of Pediatrics Mass General Brigham for Children, Harvard Medical School Boston Massachusetts USA; ^4^ Center for Global Health, Perelman School of Medicine University of Pennsylvania Philadelphia Pennsylvania USA

**Keywords:** anti‐TNF, Crohn's disease, ulcerative colitis, ustekinumab, vedolizumab

## 1 INTRODUCTION

Patients with inflammatory bowel diseases (IBD) are more susceptible to infections due to the immune dysregulation and inflammatory nature of the disease. Biologic therapies may further increase the risk, as most are immunosuppressive agents, although the degree of suppression and its association with severe infection remain uncertain. Children with IBD are a unique population, as their developing immunity and distinct disease course differ from adults.^1^ In the pediatric population, currently only tumor necrosis factor‐alpha inhibitors (TNFi) are Food and Drug Administration‐approved, while other biologics such as ustekinumab and vedolizumab are used off‐label.^2^, ^3^ Yet, their treatment outcomes have been largely extrapolated from adult studies, evaluated in small pediatric cohorts, or assessed against placebos. As biologic use expands in pediatric care, establishing their safety profiles becomes essential, especially with respect to the infection risk. To fill this knowledge gap, the present study assessed the risk of serious infection associated with biologics currently used for treating children with IBD in head‐to‐head comparative analyses.

## 2 METHODS

### 2.1 Ethics statement

This retrospective study was exempt from the requirement of informed consent. The analysis used existing, de‐identified data and involved no intervention or interaction with human subjects. Data were de‐identified in accordance with the standards outlined in Section §164.514(a) of the Health Insurance Portability and Accountability Act Privacy Rule. A qualified expert formally verified the de‐identification process, with the expert determination most recently updated in December 2020.

### 2.2 Study design and population

We analyzed de‐identified electronic health records from multiple U.S. healthcare organizations spanning October 1, 2015 to June 30, 2024. The study followed the Strengthening the Reporting of Observational Studies in Epidemiology (STROBE) reporting guidelines. Patients with IBD (Crohn's disease [CD] or ulcerative colitis [UC]) diagnosed before 18 years of age who had been biologic‐naïve were eligible for the study. The cohorts were established based on the prescriptions: TNFi (i.e., adalimumab, certolizumab, golimumab, and infliximab); immunomodulators (i.e., azathioprine, 6‐mercaptopurine, and methotrexate); ustekinumab; and vedolizumab. TNFi combination therapy (combo) was defined as the concurrent use of TNFi and immunomodulators within 3 months, while TNFi monotherapy was defined as TNFi use alone during this period. Six pairwise comparisons were conducted: (1) TNFi combo versus monotherapy; (2) ustekinumab versus TNFi monotherapy; (3) ustekinumab versus TNFi combo; (4) vedolizumab versus TNFi monotherapy; (5) vedolizumab versus TNFi combo; (6) and ustekinumab versus vedolizumab. The index date was designated as the date of the first prescription. To build the temporal relationship between the diagnosis and treatment, the diagnosis was required to be documented in the records within 1 year preceding the index date. Additional details regarding the database, data acquisition, and regulations are included in Supplemental Methods S1 and Table S2.

The baseline characteristics included demographics, comorbid diseases, prior medication use, and prior surgical history for IBD. The follow‐up duration was 3 years for this study. All patients were tracked from the day after index date until the following censoring events: occurrence of study outcomes, death, loss to follow‐up, or the end of the study period, whichever came first. The primary outcome was serious infection (defined as those requiring hospitalization, intravenous antibiotics, advanced medical intervention, or those that are life‐threatening; sepsis, opportunistic infection, central nervous system infection, lower respiratory tract infection, peritonitis, pyelonephritis, and osteomyelitis).^4^, ^5^

### 2.3 Statistical analysis

A 1:1 propensity score matching was conducted using the nearest‐neighbor algorithm, with a caliper set at 0.1 pooled standard deviations (SDs) of the logit of the propensity score, to balance baseline covariates between the comparison cohorts. The Poisson regression model was used to calculate the risk ratios (RRs) with 95% confidence intervals (CIs). Subgroup analyses were performed based on the infectious categories, including gastrointestinal, urinary tract, respiratory, central nervous system, opportunistic infections, and sepsis; and IBD subtypes, including CD and UC. Cases classified as indeterminant colitis or with unspecified IBD were not included. All statistical analyses were conducted on the built‐in Advanced Analytics in TriNetX research platform and RStudio (Version: 2023.12.1 + 402).

## 3 RESULTS

We included a total of 15,976 pediatric patients with IBD in the cohort of TNFi monotherapy, 3152 with TNFi combo, 2074 with ustekinumab, and 1561 with vedolizumab before matching. After 1:1 propensity score matching, the following pairs were compared: 3142 (TNFi combo vs. TNFi monotherapy), 1936 (ustekinumab vs. TNFi monotherapy), 1887 (ustekinumab vs. TNFi combo), 1549 (vedolizumab vs. TNFi monotherapy), 1510 (vedolizumab vs. TNFi combo), and 1406 (ustekinumab vs. vedolizumab). The mean age of the patients with IBD initiating treatments ranged from 13.7 to 14.7 years old. The baseline characteristics for each comparison after propensity score matching are presented in Tables [Link], [Link], [Link], [Link], [Link], [Link].

### 3.1 Serious infection

TNFi combo was associated with a higher risk of serious infection than monotherapy (13.1% vs. 10.8%, RR: 1.22 [95% CI: 1.05–1.41]). Ustekinumab was associated with a lower infection risk compared to both TNFi monotherapy (9.1% vs. 12.6%, RR: 0.72 [95% CI: 0.58–0.88]) and combo (9.2% vs. 12.7%, RR: 0.72 [95% CI: 0.59–0.89]). In contrast, vedolizumab showed a comparable infection risk to TNFi therapies, with or without combinations.

**Figure 1:**
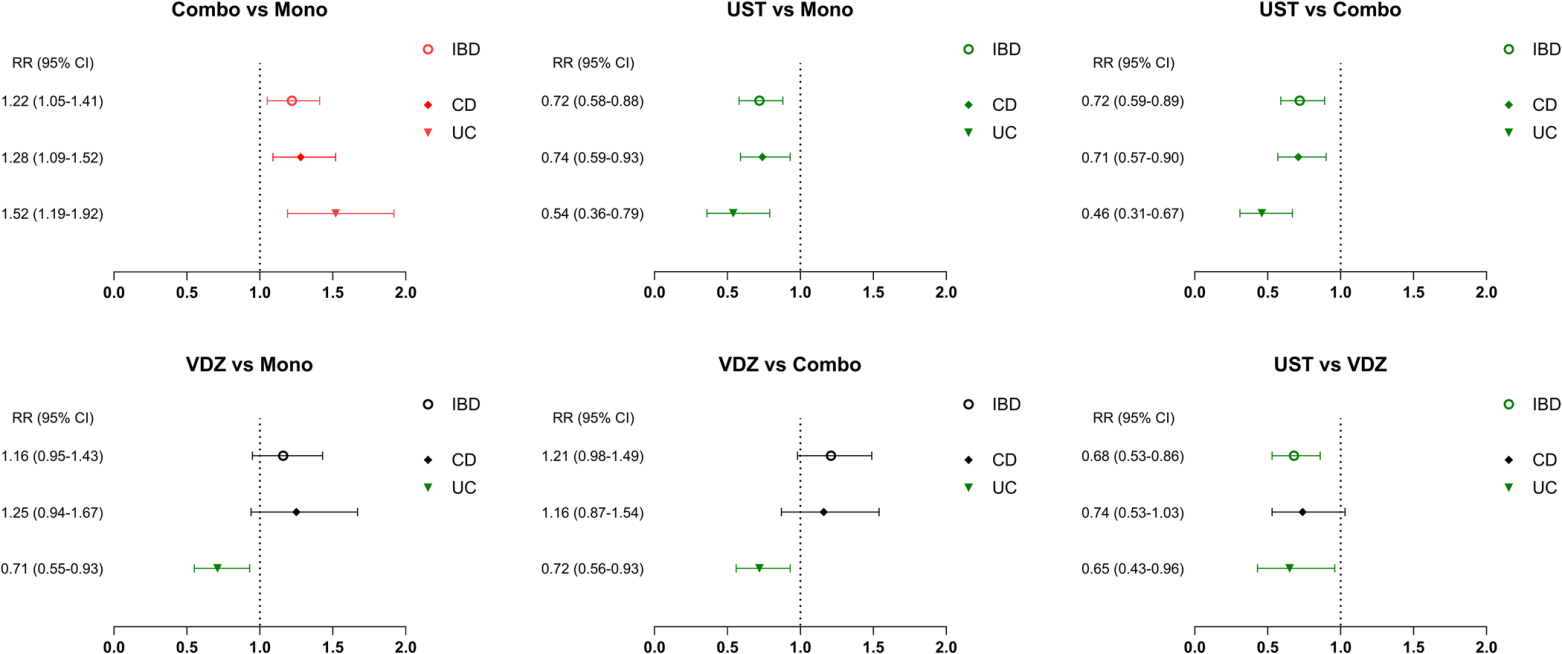
Comparative serious infection risks of biologics in pediatric patients with IBD (by IBD subtypes). CD, Crohn's disease; CI, confidence interval; IBD, inflammatory bowel disease; Mono, TNFi monotherapy; UC, ulcerative colitis; UST, ustekinumab; VDZ, vedolizumab.

Ustekinumab was associated with a lower infection risk when compared with vedolizumab (9.2% vs. 13.6%, RR: 0.68 [95% CI: 0.53–0.86]; Figure 1).

### 3.2 Subgroup analysis: IBD subtypes

Subgroup analyses by IBD subtype generally aligned with the primary findings, with some variation observed for vedolizumab: (1) in UC, vedolizumab was associated with a lower risk of serious infection compared to TNFi therapies; and (2) in CD, vedolizumab showed a comparable infection risk to ustekinumab (Figure 1).

### 3.3 Subgroup analysis: Infectious categories

**Figure 2:**
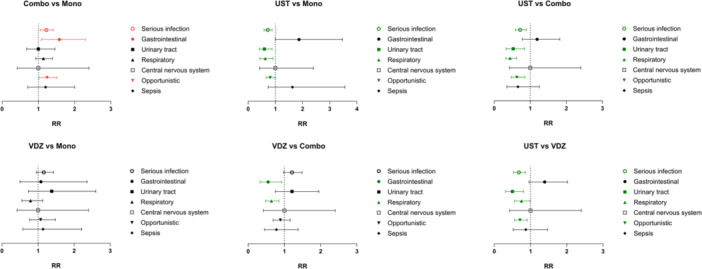
Comparative infection risks of biologics in pediatric patients with IBD (by infectious categories). Risk ratios are color‐coded based on statistical significance and direction of effect. Significant findings with RR < 1 are shown in green, indicating a lower risk in the comparator group (left) relative to the reference group (right). Significant findings with RR > 1 are shown in red, indicating a higher risk in the comparator group relative to the reference group. Combo, TNFi combination therapy; Mono, TNFi monotherapy; RR, risk ratio; UST, ustekinumab; VDZ, vedolizumab.

Significant differences in infection risk were primarily observed in opportunistic and respiratory tract infections across treatment comparisons, followed by gastrointestinal and urinary tract infections (Figure 2 and Table S9).

## 4 DISCUSSION

To the best of our knowledge, there have been no head‐to‐head comparisons of safety profiles, specifically regarding the risk of serious infection, among biologics for treating IBD in the pediatric population. As a result, we referred extensively to findings from previous adult studies for comparison with our pediatric data.

In pediatric patients with IBD, our data revealed an elevated risk of serious infection, particularly opportunistic infection, associated with the concomitant use of immunosuppressive agents with TNFi therapy. This observation aligns with prior reports in adults, where combination therapy (TNFi plus thiopurines) increased serious and opportunistic infection risk compared to TNFi monotherapy.^6^ In those studies, the excess risk was largely attributed to thiopurines, which, however, are increasingly being replaced by biologics and are less frequently used in current practice.^7^

A recent meta‐analysis found that vedolizumab was associated with a 32% lower odds of serious infection versus TNFi in adult patients with UC, while no significant difference was found between these two agents in the overall population or in CD specifically.^8^ Similarly, we reported a 29% lower risk of serious infection with vedolizumab versus TNFi in pediatric patients with UC, and nonsignificant results in the overall IBD and CD populations. Several theories may explain these findings, with one common hypothesis suggesting that vedolizumab is more effective in UC than in CD for achieving disease control, a trend also reported in pediatric data.^9^ Vedolizumab failure in patients with CD, which in turn may lead to increased use of corticosteroids, could subsequently contribute to a higher risk of serious infection.^10^

A large US cohort study reported that ustekinumab was associated with a reduced infectious risk compared to TNFi in adult patients with IBD.^11^ The prior meta‐analysis also showed that patients with CD treated with ustekinumab had significantly lower odds of serious infection compared to those treated with TNFi, while data on UC remained limited.^8^ Our findings concurred with the results of these studies and further supported the lower infection risk of ustekinumab compared to TNFi in the pediatric IBD population, independent of disease subtypes.

Head‐to‐head comparisons of treatment outcomes between ustekinumab and vedolizumab have been limited, with the findings in the literature being conflicting. In the meta‐analysis, ustekinumab was associated with a 60% lower risk of serious infection compared to vedolizumab in adult patients with CD.^8^ In contrast, a real‐world study demonstrated that no significant difference in rates of opportunistic or serious infection‐related encounters was observed between adult patients with CD treated with vedolizumab and ustekinumab, which aligns more closely with our pediatric data.^12^ A retrospective cohort study reported that vedolizumab‐treated patients had a higher risk of gastrointestinal infection than those treated with ustekinumab.^13^ In our cohort, ustekinumab was similarly associated with less serious infection than vedolizumab, although our data did not replicate the previously reported difference in gastrointestinal infection risk.

The current study presents several strengths. The large sample size enhances statistical power and enables the detection of rare events. There are also some limitations. First, the evidence is limited due to the retrospective and observational nature of the study. Second, the use of billing codes might introduce misclassification bias. Third, we were unable to extract detailed information on disease severity, phenotypes, laboratory biomarkers, disease duration, time to the first treatment, treatment dosage and duration, and other adjunctive medications (e.g., steroids). Fourth, despite a rigorous matching process, unmeasured confounding may still exist, leading to selection bias. Fifth, our dataset consists of about 70% White individuals, which may limit the generalizability of the results. Lastly, the study selected categories of serious infection based on clinical relevance, prior reports, and feasibility within the dataset and may not capture the full spectrum of serious infectious events.

## 5 CONCLUSIONS

In this comparative study of serious infection risk among different biologic agents for treating pediatric patients with IBD, TNFi combo was associated with a higher risk of serious infection, while ustekinumab showed lower risks of serious infection when compared to other treatments. Long‐term, prospective safety registries are needed to confirm these associations and to support the expedited approval of medications currently being used off‐label to treat children with CD and UC.

## CONFLICTS OF INTEREST STATEMENT

Ashwin N. Ananthakrishnan: Consultant: Geneoscopy. Harland S. Winter: Consultant: AbbVie, Janssen Pharmaceuticals, Pfizer, Nestle, Women's Wellness Foundation, Autism Research Institute, Pediatric IBD Foundation. The remaining authors declare no conflict of interest.

## Supporting information

Supplemental Methods.

suppTable1.

suppTable2.

suppTable3.

suppTable4.

suppTable5.

suppTable6.

suppTable7.

suppTable8.

## Data Availability

Data are available on request by contacting the corresponding author.
