# Supplementary material for: Comparative risk of serious infection among biologic therapies for inflammatory bowel disease in pediatric patients: A target trial emulation
Source: J Pediatr Gastroenterol Nutr. 2025 Nov 25;82(2):503–7. doi: 10.1002/jpn3.70251 (PMC12864173; doi:10.1002/jpn3.70251)
Supplement: Supplementary file 1 — Supplemental Methods. [file JPN3-82-503-s007.docx]

**Supplemental Digital Content 1.** Supplemental Methods

This population-based cohort study adopted the target trial emulation framework to mimic a randomized clinical trial. All data were retrieved from the TriNetX Research Network (TriNetX, LLC, Cambridge, Massachusetts, USA). The platform collects de-identified electronic health records from its collaborating healthcare organizations (HCOs) across the nation. The HCOs are primarily large academic centers providing both tertiary care and outpatient services through satellite locations. TriNetX maps data from electronic health records, laboratory results, and registries to standardized, curated clinical terminologies and then converts them into a proprietary data structure. The platform performs thorough quality checks and removes records that do not meet data quality standards during the data-cleaning process. The data include patients who are both insured and uninsured. The platform enables researchers to construct queries using prespecified inclusion and exclusion criteria. Diagnoses and treatments are retrieved using standardized coding systems, including the International Classification of Diseases, Tenth Revision, Clinical Modification (ICD-10-CM), Anatomical Therapeutic Chemical (ATC), RxNorm, and Healthcare Common Procedure Coding System (HCPCS) codes. The specific codes used in this study are provided in Supplementary Table 1. Data from October 1, 2015 through June 30, 2024 that met the eligibility criteria were extracted from the network. All analyses were performed using the built-in analytics.
