# Supplementary material for: Comparative risk of serious infection among biologic therapies for inflammatory bowel disease in pediatric patients: A target trial emulation
Source: J Pediatr Gastroenterol Nutr. 2025 Nov 25;82(2):503–7. doi: 10.1002/jpn3.70251 (PMC12864173; doi:10.1002/jpn3.70251)
Supplement: Supplementary file 2 — suppTable1. [file JPN3-82-503-s003.docx]

**Table S1**. Standardized names and codes of variables (exposures, covariates, and outcomes)

|  | ICD-10, CPT, RxNorm, ATC, HSPCS |
| --- | --- |
| Inflammatory bowel disease | ICD-10: K50, K51 |
| Crohn’s disease | ICD-10: K50 |
| Ulcerative colitis | ICD-10: K51 |
| Medications |  |
| Systemic corticosteroids | ATC: H02 |
| TNF inhibitors (adalimumab, certolizumab, golimumab, and infliximab) | RxNorm: 327361, 709271, 819300, 191831  HCPCS: J0135, J0717, J1602, J1745 |
| Immunomodulators (azathioprine, 6-mercaptopurine, and methotrexate) | RxNorm: 1256, 103, 6851  HCPCS: J7500, J7501, S0108, J9250, J9260, J8610 |
| Biologics other than TNF inhibitors (ustekinumab, vedolizumab, and risankizumab) | ATC: L04AC05, L04AG05, L04AC18 |
| Vedolizumab | RxNorm: 1538097, 80280  HCPCS: J3380, C9026  ATC: L04AG05 |
| Ustekinumab | RxNorm: 847083  HCPCS: J3358, J3357, Q9989, C9487  ATC: L04AC05 |
| Baseline characteristics |  |
| Hypertension | ICD-10: I10-I1A |
| Type 1 diabetes mellitus | ICD-10: E10 |
| Type 2 diabetes mellitus | ICD-10: E11 |
| Metabolic syndrome | ICD-10: E70-E88 |
| Celiac disease | ICD-10: K90.0 |
| Autoimmune hepatitis | ICD-10: K75.4 |
| Autoimmune thyroiditis | ICD-10: E06.3 |
| Systemic lupus erythematous | ICD-10: M32 |
| Psoriasis | ICD-10: L40 |
| Inflammatory polyarthropathies | ICD-10: M05-M14 |
| Asthma | ICD-10: J45 |
| Procedures |  |
| Resection of small bowel | HSPCS: 44120, 44121, 44125, 44126, 44127, 44128, 44202 |
| Ileocolic resection or right-sided hemicolectomy | HSPCS: 44160, 44205 |
| Colectomy | HSPCS: 44140, 44141, 44143, 44144, 44145, 44146, 44147, 44150, 44151, 44155, 44156, 44157, 44158, 44320, 44322 |
| Proctectomy | HSPCS: 45110, 45112, 45113, 45114, 45119, 45120, 45121, 45123 |
| Laparotomy | HSPCS: 44204, 44205, 44206, 44207, 44208, 44210, 44211, 44212, 44213, 44238, 45395, 45397, 45499 |
| Diagnostic outcomes |  |
| Serious infection (sepsis, opportunistic infections, central nervous system infections, lower respiratory tract infection, peritonitis, pyelonephritis, and osteomyelitis) | ICD-10: A00-A09, A15-A19, A22.7, A26.7, A32.7, A40, A41, A42.7, A54.86, B01-B02, B25, B35-B49, B50-B64, B65-B83, G00-G09, J09-J18, J20-J22, K56, M86, N10-N12, R65.2 |
| Gastrointestinal infection | ICD-10: A00-A09 |
| Urinary tract infection | ICD-10: N10-N12 |
| Respiratory infection | ICD-10: J09-J18, J20-J22 |
| Central nervous system infection | ICD-10: G00-G09 |
| Opportunistic infection | ICD-10: A15-A19, A22.7, A26.7, A32.7, A40, A41, A42.7, A54.86, B01-B02, B25, B35-B49, B50-B64, B65-B83 |
| Sepsis | ICD-10: R65.2 |

ATC, Anatomical Therapeutic Chemical; HCPCS, Healthcare Common Procedure Coding System; ICD-10, International Classification of Diseases, Tenth Revision; TNF, tumor necrosis factor
