# Supplementary material for: Comparative risk of serious infection among biologic therapies for inflammatory bowel disease in pediatric patients: A target trial emulation
Source: J Pediatr Gastroenterol Nutr. 2025 Nov 25;82(2):503–7. doi: 10.1002/jpn3.70251 (PMC12864173; doi:10.1002/jpn3.70251)
Supplement: Supplementary file 3 — suppTable2. [file JPN3-82-503-s006.docx]

**Table S2.** Baseline characteristics in anti-TNF combination therapy versus anti-TNF monotherapy in pediatric patients with IBD

|  | Anti-TNF combination therapy  (n=3,142) | Anti-TNF monotherapy  (n=3,142) | SMD |
| --- | --- | --- | --- |
| Age at index, mean ± SD (years) | 13.7 ± 3.4 | 13.7 ± 3.4 | <0.001 |
| Follow-up, median (IQR, years) | 3.0 (0.4) | 3.0 (1.5) | — |
| Sex, n (%) |  |  |  |
| Female | 1,487 (47.3) | 1,485 (47.3) | 0.001 |
| Race, n (%) |  |  |  |
| White | 2,283 (72.7) | 2,284 (72.7) | 0.001 |
| Black or African American | 324 (10.3) | 323 (10.3) | 0.001 |
| Asian | 132 (4.2) | 132 (4.2) | <0.001 |
| Native Hawaiian or other Pacific Islander | ≤10 (0.3) | ≤10 (0.3) | <0.001 |
| American Indian or Alaska Native | ≤10 (0.3) | ≤10 (0.3) | <0.001 |
| Other | 146 (4.6) | 148 (4.7) | 0.003 |
| Unknown | 243 (7.7) | 244 (7.8) | 0.001 |
| Comorbid condition, n (%) |  |  |  |
| Hypertension | 64 (2.0) | 63 (2.0) | 0.002 |
| Type 1 diabetes mellitus | 19 (0.6) | 19 (0.6) | <0.001 |
| Type 2 diabetes mellitus | 25 (0.8) | 24 (0.8) | 0.004 |
| Metabolic syndrome | 848 (27.0) | 845 (26.9) | 0.002 |
| Celiac disease | 56 (1.8) | 56 (1.8) | <0.001 |
| Autoimmune hepatitis | 21 (0.7) | 21 (0.7) | <0.001 |
| Autoimmune thyroiditis | 14 (0.4) | 16 (0.5) | 0.009 |
| Systemic lupus erythematous | ≤10 (0.3) | ≤10 (0.3) | <0.001 |
| Psoriasis | 52 (1.7) | 51 (1.6) | 0.003 |
| Inflammatory polyarthropathies | 92 (2.9) | 89 (2.8) | 0.006 |
| Asthma | 353 (11.2) | 354 (11.3) | 0.001 |
| Prior use of medication, n (%) |  |  |  |
| Systemic corticosteroids | 2,131 (67.8) | 2,130 (67.8) | 0.001 |
| Immunomodulators | — | — | — |
| TNF-alpha inhibitors | — | — | — |
| Biologics other than TNF inhibitors | 17 (0.5) | 17 (0.5) | <0.001 |
| Prior surgical history, n (%) |  |  |  |
| Resection of small bowel | ≤10 (0.3) | ≤10 (0.3) | <0.001 |
| Ileocolic resection or right-sided hemicolectomy | ≤10 (0.3) | ≤10 (0.3) | <0.001 |
| Colectomy | ≤10 (0.3) | ≤10 (0.3) | <0.001 |
| Proctectomy | ≤10 (0.3) | ≤10 (0.3) | <0.001 |
| Laparotomy | ≤10 (0.3) | ≤10 (0.3) | <0.001 |

SD, standard deviation; SMD, standardized mean difference; IBD, inflammatory bowel diseases; IQR, interquartile range; TNF, tumor necrosis factor

*An em dash indicates unavailable data because the variable represents the exposure itself and therefore was not included in the matching process.
