# Supplementary material for: Comparative risk of serious infection among biologic therapies for inflammatory bowel disease in pediatric patients: A target trial emulation
Source: J Pediatr Gastroenterol Nutr. 2025 Nov 25;82(2):503–7. doi: 10.1002/jpn3.70251 (PMC12864173; doi:10.1002/jpn3.70251)
Supplement: Supplementary file 4 — suppTable3. [file JPN3-82-503-s002.docx]

**Table S3**. Baseline characteristics in ustekinumab versus anti-TNF monotherapy in pediatric patients with IBD

|  | Ustekinumab  (n=1,936) | Anti-TNF monotherapy  (n=1,936) | SMD |
| --- | --- | --- | --- |
| Age at index, mean ± SD (years) | 14.6 ± 3.3 | 14.7 ± 3.2 | 0.016 |
| Follow-up, median (IQR, years) | 1.9 (2.1) | 3.0 (1.3) | — |
| Sex, n (%) |  |  |  |
| Female | 915 (47.3) | 927 (47.9) | 0.012 |
| Race, n (%) |  |  |  |
| White | 1,370 (70.8) | 1,380 (71.3) | 0.011 |
| Black or African American | 193 (10.0) | 193 (10.0) | <0.001 |
| Asian | 91 (4.7) | 97 (5.0) | 0.014 |
| Native Hawaiian or other Pacific Islander | ≤10 (0.5) | ≤10 (0.5) | <0.001 |
| American Indian or Alaska Native | ≤10 (0.5) | ≤10 (0.5) | <0.001 |
| Other | 97 (5.0) | 84 (4.3) | 0.032 |
| Unknown | 182 (9.4) | 176 (9.1) | 0.011 |
| Comorbid condition, n (%) |  |  |  |
| Hypertension | 85 (4.4) | 72 (3.7) | 0.002 |
| Type 1 diabetes mellitus | 16 (0.8) | ≤10 (0.5) | 0.038 |
| Type 2 diabetes mellitus | 29 (1.5) | 25 (1.3) | 0.018 |
| Metabolic syndrome | 739 (38.2) | 686 (35.4) | 0.057 |
| Celiac disease | 54 (2.8) | 52 (2.7) | 0.006 |
| Autoimmune hepatitis | 22 (1.1) | 22 (1.1) | <0.001 |
| Autoimmune thyroiditis | 16 (0.8) | ≤10 (0.5) | 0.038 |
| Systemic lupus erythematous | ≤10 (0.5) | ≤10 (0.5) | <0.001 |
| Psoriasis | 118 (6.1) | 120 (6.2) | 0.004 |
| Inflammatory polyarthropathies | 103 (5.3) | 108 (5.6) | 0.011 |
| Asthma | 300 (15.5) | 303 (15.7) | 0.004 |
| Prior use of medication, n (%) |  |  |  |
| Systemic corticosteroids | 1,491 (77.0) | 1,467 (75.8) | 0.029 |
| Immunomodulators | 1,459 (75.4) | 1,455 (75.2) | 0.005 |
| TNF-alpha inhibitors | — | — | — |
| Biologics other than TNF inhibitors | — | — | — |
| Prior surgical history, n (%) |  |  |  |
| Resection of small bowel | ≤10 (0.5) | ≤10 (0.5) | <0.001 |
| Ileocolic resection or right-sided hemicolectomy | ≤10 (0.5) | ≤10 (0.5) | <0.001 |
| Colectomy | ≤10 (0.5) | ≤10 (0.5) | <0.001 |
| Proctectomy | ≤10 (0.5) | ≤10 (0.5) | <0.001 |
| Laparotomy | ≤10 (0.5) | ≤10 (0.5) | <0.001 |

SD, standard deviation; SMD, standardized mean difference; IBD, inflammatory bowel diseases; IQR, interquartile range; TNF, tumor necrosis factor

*An em dash indicates unavailable data because the variable represents the exposure itself and therefore was not included in the matching process.
