# Supplementary material for: Comparative risk of serious infection among biologic therapies for inflammatory bowel disease in pediatric patients: A target trial emulation
Source: J Pediatr Gastroenterol Nutr. 2025 Nov 25;82(2):503–7. doi: 10.1002/jpn3.70251 (PMC12864173; doi:10.1002/jpn3.70251)
Supplement: Supplementary file 5 — suppTable4. [file JPN3-82-503-s008.docx]

**Table S4**. Baseline characteristics in ustekinumab versus anti-TNF combination therapy in pediatric patients with IBD

|  | Ustekinumab  (n=1,887) | Anti-TNF combination therapy  (n=1,887) | SMD |
| --- | --- | --- | --- |
| Age at index, mean ± SD (years) | 14.6 ± 3.3 | 14.7 ± 3.1 | 0.020 |
| Follow-up, median (IQR, years) | 1.9 (2.1) | 3.0 (0.6) | — |
| Sex, n (%) |  |  |  |
| Female | 895 (47.4) | 911 (48.3) | 0.017 |
| Race, n (%) |  |  |  |
| White | 1,331 (70.5) | 1,331 (70.5) | <0.001 |
| Black or African American | 185 (9.8) | 197 (10.4) | 0.021 |
| Asian | 88 (4.7) | 91 (4.8) | 0.007 |
| Native Hawaiian or other Pacific Islander | ≤10 (0.5) | ≤10 (0.5) | <0.001 |
| American Indian or Alaska Native | ≤10 (0.5) | ≤10 (0.5) | <0.001 |
| Other | 97 (5.1) | 78 (4.1) | 0.048 |
| Unknown | 182 (9.6) | 182 (9.6) | <0.001 |
| Comorbid condition, n (%) |  |  |  |
| Hypertension | 61 (3.2) | 55 (2.9) | 0.018 |
| Type 1 diabetes mellitus | 15 (0.8) | 14 (0.7) | 0.006 |
| Type 2 diabetes mellitus | 20 (1.0) | 21 (1.1) | 0.005 |
| Metabolic syndrome | 670 (35.5) | 673 (35.7) | 0.003 |
| Celiac disease | 49 (2.6) | 37 (2.0) | 0.043 |
| Autoimmune hepatitis | 22 (1.2) | 16 (0.8) | 0.032 |
| Autoimmune thyroiditis | 14 (0.7) | 12 (0.6) | 0.013 |
| Systemic lupus erythematous | ≤10 (0.5) | ≤10 (0.5) | <0.001 |
| Psoriasis | 56 (3.0) | 53 (2.8) | 0.009 |
| Inflammatory polyarthropathies | 83 (4.4) | 78 (4.1) | 0.013 |
| Asthma | 289 (15.3) | 286 (15.2) | 0.004 |
| Prior use of medication, n (%) |  |  |  |
| Systemic corticosteroids | 1,440 (76.3) | 1,449 (76.8) | 0.011 |
| Immunomodulators | — | — | — |
| TNF-alpha inhibitors | — | — | — |
| Biologics other than TNF inhibitors | — | — | — |
| Prior surgical history, n (%) |  |  |  |
| Resection of small bowel | ≤10 (0.5) | ≤10 (0.5) | <0.001 |
| Ileocolic resection or right-sided hemicolectomy | ≤10 (0.5) | ≤10 (0.5) | <0.001 |
| Colectomy | ≤10 (0.5) | ≤10 (0.5) | <0.001 |
| Proctectomy | ≤10 (0.5) | ≤10 (0.5) | <0.001 |
| Laparotomy | ≤10 (0.5) | ≤10 (0.5) | <0.001 |

SD, standard deviation; SMD, standardized mean difference; IBD, inflammatory bowel diseases; IQR, interquartile range; TNF, tumor necrosis factor

*An em dash indicates unavailable data because the variable represents the exposure itself and therefore was not included in the matching process.
