# Supplementary material for: Comparative risk of serious infection among biologic therapies for inflammatory bowel disease in pediatric patients: A target trial emulation
Source: J Pediatr Gastroenterol Nutr. 2025 Nov 25;82(2):503–7. doi: 10.1002/jpn3.70251 (PMC12864173; doi:10.1002/jpn3.70251)
Supplement: Supplementary file 6 — suppTable5. [file JPN3-82-503-s004.docx]

**Table S5**. Baseline characteristics in vedolizumab versus anti-TNF monotherapy in pediatric patients with IBD

|  | Vedolizumab  (n=1,549) | Anti-TNF monotherapy  (n=1,549) | SMD |
| --- | --- | --- | --- |
| Age at index, mean ± SD (years) | 14.4 ± 3.7 | 14.5 ± 3.5 | 0.027 |
| Follow-up, median (IQR, years) | 2.8 (1.7) | 3.0 (1.3) | — |
| Sex, n (%) |  |  |  |
| Female | 733 (47.3) | 729 (47.1) | 0.005 |
| Race, n (%) |  |  |  |
| White | 1,102 (71.1) | 1,105 (71.3) | 0.004 |
| Black or African American | 134 (8.7) | 123 (7.9) | 0.026 |
| Asian | 56 (3.6) | 61 (3.9) | 0.017 |
| Native Hawaiian or other Pacific Islander | ≤10 (0.6) | ≤10 (0.6) | <0.001 |
| American Indian or Alaska Native | 11 (0.7) | 11 (0.7) | <0.001 |
| Other | 97 (6.3) | 85 (5.5) | 0.033 |
| Unknown | 147 (9.5) | 163 (10.5) | 0.034 |
| Comorbid condition, n (%) |  |  |  |
| Hypertension | 81 (5.2) | 79 (5.1) | 0.006 |
| Type 1 diabetes mellitus | ≤10 (0.6) | ≤10 (0.6) | <0.001 |
| Type 2 diabetes mellitus | 31 (2.0) | 23 (1.5) | 0.039 |
| Metabolic syndrome | 575 (37.1) | 551 (35.6) | 0.032 |
| Celiac disease | 40 (2.6) | 34 (2.2) | 0.025 |
| Autoimmune hepatitis | 25 (1.6) | 20 (1.3) | 0.027 |
| Autoimmune thyroiditis | 13 (0.8) | 11 (0.7) | 0.015 |
| Systemic lupus erythematous | ≤10 (0.6) | 11 (0.7) | 0.008 |
| Psoriasis | 72 (4.6) | 71 (4.6) | 0.003 |
| Inflammatory polyarthropathies | 53 (3.4) | 45 (2.9) | 0.030 |
| Asthma | 229 (14.8) | 229 (14.8) | <0.001 |
| Prior use of medication, n (%) |  |  |  |
| Systemic corticosteroids | 1,165 (75.2) | 1,147 (74.0) | 0.027 |
| Immunomodulators | 1,049 (67.7) | 1,048 (67.7) | 0.001 |
| TNF-alpha inhibitors | — | — | — |
| Biologics other than TNF inhibitors | — | — | — |
| Prior surgical history, n (%) |  |  |  |
| Resection of small bowel | ≤10 (0.6) | ≤10 (0.6) | <0.001 |
| Ileocolic resection or right-sided hemicolectomy | ≤10 (0.6) | ≤10 (0.6) | <0.001 |
| Colectomy | ≤10 (0.6) | ≤10 (0.6) | <0.001 |
| Proctectomy | ≤10 (0.6) | ≤10 (0.6) | <0.001 |
| Laparotomy | ≤10 (0.6) | ≤10 (0.6) | <0.001 |

SD, standard deviation; SMD, standardized mean difference; IBD, inflammatory bowel diseases; IQR, interquartile range; TNF, tumor necrosis factor

*An em dash indicates unavailable data because the variable represents the exposure itself and therefore was not included in the matching process.
