# Supplementary material for: Comparative risk of serious infection among biologic therapies for inflammatory bowel disease in pediatric patients: A target trial emulation
Source: J Pediatr Gastroenterol Nutr. 2025 Nov 25;82(2):503–7. doi: 10.1002/jpn3.70251 (PMC12864173; doi:10.1002/jpn3.70251)
Supplement: Supplementary file 7 — suppTable6. [file JPN3-82-503-s005.docx]

**Table S6**. Baseline characteristics in vedolizumab versus anti-TNF combination therapy in pediatric patients with IBD

|  | Vedolizumab  (n=1,510) | Anti-TNF combination therapy  (n=1,510) | SMD |
| --- | --- | --- | --- |
| Age at index, mean ± SD (years) | 14.3 ± 3.7 | 14.4 ± 3.4 | 0.021 |
| Follow-up, median (IQR, years) | 2.8 (1.7) | 3.0 (0.3) | — |
| Sex, n (%) |  |  |  |
| Female | 714 (47.3) | 705 (46.7) | 0.012 |
| Race, n (%) |  |  |  |
| White | 1,074 (71.1) | 1,076 (71.3) | 0.003 |
| Black or African American | 130 (8.6) | 138 (9.1) | 0.019 |
| Asian | 56 (3.7) | 49 (3.2) | 0.025 |
| Native Hawaiian or other Pacific Islander | ≤10 (0.7) | ≤10 (0.7) | <0.001 |
| American Indian or Alaska Native | ≤10 (0.7) | ≤10 (0.7) | <0.001 |
| Other | 92 (6.1) | 82 (5.4) | 0.028 |
| Unknown | 146 (9.7) | 157 (10.4) | 0.024 |
| Comorbid condition, n (%) |  |  |  |
| Hypertension | 60 (4.0) | 58 (3.8) | 0.007 |
| Type 1 diabetes mellitus | ≤10 (0.7) | ≤10 (0.7) | <0.001 |
| Type 2 diabetes mellitus | 21 (1.4) | 20 (1.3) | 0.006 |
| Metabolic syndrome | 544 (36.0) | 554 (36.7) | 0.014 |
| Celiac disease | 40 (2.6) | 34 (2.2) | 0.025 |
| Autoimmune hepatitis | 20 (1.3) | 17 (1.1) | 0.018 |
| Autoimmune thyroiditis | 11 (0.7) | 12 (0.8) | 0.008 |
| Systemic lupus erythematous | ≤10 (0.7) | ≤10 (0.7) | <0.001 |
| Psoriasis | 45 (3.0) | 46 (3.0) | 0.004 |
| Inflammatory polyarthropathies | 47 (3.1) | 37 (2.5) | 0.040 |
| Asthma | 216 (14.3) | 222 (14.7) | 0.011 |
| Prior use of medication, n (%) |  |  |  |
| Systemic corticosteroids | 1,132 (75.0) | 1,121 (74.2) | 0.017 |
| Immunomodulators | — | — | — |
| TNF-alpha inhibitors | — | — | — |
| Biologics other than TNF inhibitors | — | — | — |
| Prior surgical history, n (%) |  |  |  |
| Resection of small bowel | ≤10 (0.7) | ≤10 (0.7) | <0.001 |
| Ileocolic resection or right-sided hemicolectomy | ≤10 (0.7) | ≤10 (0.7) | <0.001 |
| Colectomy | ≤10 (0.7) | ≤10 (0.7) | <0.001 |
| Proctectomy | ≤10 (0.7) | ≤10 (0.7) | <0.001 |
| Laparotomy | ≤10 (0.7) | ≤10 (0.7) | <0.001 |

SD, standard deviation; SMD, standardized mean difference; IBD, inflammatory bowel diseases; IQR, interquartile range; TNF, tumor necrosis factor

*An em dash indicates unavailable data because the variable represents the exposure itself and therefore was not included in the matching process.
