# Supplementary material for: Comparative risk of serious infection among biologic therapies for inflammatory bowel disease in pediatric patients: A target trial emulation
Source: J Pediatr Gastroenterol Nutr. 2025 Nov 25;82(2):503–7. doi: 10.1002/jpn3.70251 (PMC12864173; doi:10.1002/jpn3.70251)
Supplement: Supplementary file 8 — suppTable7. [file JPN3-82-503-s001.docx]

**Table S7.** Baseline characteristics in ustekinumab versus vedolizumab in pediatric patients with IBD

|  | Ustekinumab  (n=1,406) | Vedolizumab  (n=1,406) | SMD |
| --- | --- | --- | --- |
| Age at index, mean ± SD (years) | 14.5 ± 3.4 | 14.4 ± 3.6 | 0.023 |
| Follow-up, median (IQR, years) | 2.0 (2.1) | 2.8 (1.7) | — |
| Sex, n (%) |  |  |  |
| Female | 672 (47.8) | 666 (47.4) | 0.009 |
| Race, n (%) |  |  |  |
| White | 1,013 (72.0) | 996 (70.8) | 0.027 |
| Black or African American | 114 (8.1) | 128 (9.1) | 0.036 |
| Asian | 47 (3.3) | 53 (3.8) | 0.023 |
| Native Hawaiian or other Pacific Islander | ≤10 (0.7) | ≤10 (0.7) | <0.001 |
| American Indian or Alaska Native | ≤10 (0.7) | ≤10 (0.7) | <0.001 |
| Other | 78 (5.5) | 89 (6.3) | 0.033 |
| Unknown | 152 (10.8) | 129 (9.2) | 0.055 |
| Comorbid condition, n (%) |  |  |  |
| Hypertension | 64 (4.6) | 70 (5.0) | 0.020 |
| Type 1 diabetes mellitus | ≤10 (0.7) | ≤10 (0.7) | <0.001 |
| Type 2 diabetes mellitus | 27 (1.9) | 21 (1.5) | 0.033 |
| Metabolic syndrome | 543 (38.6) | 546 (38.8) | 0.004 |
| Celiac disease | 34 (2.4) | 40 (2.8) | 0.027 |
| Autoimmune hepatitis | 18 (1.2) | 20 (1.4) | 0.012 |
| Autoimmune thyroiditis | 11 (0.8) | 14 (1.0) | 0.023 |
| Systemic lupus erythematous | ≤10 (0.7) | ≤10 (0.7) | <0.001 |
| Psoriasis | 81 (5.8) | 83 (5.9) | 0.006 |
| Inflammatory polyarthropathies | 65 (4.6) | 52 (3.7) | 0.046 |
| Asthma | 206 (14.7) | 222 (15.8) | 0.003 |
| Prior use of medication, n (%) |  |  |  |
| Systemic corticosteroids | 1,096 (78.0) | 1,058 (75.2) | 0.064 |
| Immunomodulators | 1,028 (73.1) | 1,039 (73.9) | 0.018 |
| TNF-alpha inhibitors | 909 (64.7) | 915 (65.1) | 0.009 |
| Biologics other than TNF inhibitors | — | — | — |
| Prior surgical history, n (%) |  |  |  |
| Resection of small bowel | ≤10 (0.7) | ≤10 (0.7) | <0.001 |
| Ileocolic resection or right-sided hemicolectomy | ≤10 (0.7) | ≤10 (0.7) | <0.001 |
| Colectomy | ≤10 (0.7) | ≤10 (0.7) | <0.001 |
| Proctectomy | ≤10 (0.7) | ≤10 (0.7) | <0.001 |
| Laparotomy | ≤10 (0.7) | ≤10 (0.7) | <0.001 |

SD, standard deviation; SMD, standardized mean difference; IBD, inflammatory bowel diseases; IQR, interquartile range; TNF, tumor necrosis factor

*An em dash indicates unavailable data because the variable represents the exposure itself and therefore was not included in the matching process.
