# Supplementary material for: Comparative risk of serious infection among biologic therapies for inflammatory bowel disease in pediatric patients: A target trial emulation
Source: J Pediatr Gastroenterol Nutr. 2025 Nov 25;82(2):503–7. doi: 10.1002/jpn3.70251 (PMC12864173; doi:10.1002/jpn3.70251)
Supplement: Supplementary file 9 — suppTable8. [file JPN3-82-503-s009.docx]

**Table S8.** Risk ratios of pairwise comparisons of biologics in pediatric patients with IBD (by infectious categories)

|  | RR (95% CI) | | | | | | |
| --- | --- | --- | --- | --- | --- | --- | --- |
|  | Serious infection | Gastrointestinal | Urinary tract | Respiratory | Central nervous system | Opportunistic | Sepsis |
| **Combo vs Mono** |  |  |  |  |  |  |  |
| Combo | 1.22 (1.05-1.41) | 1.58 (1.09-2.30) | 1.00 (0.68-1.46) | 1.14 (0.93-1.39) | 1.00 (0.42-2.40) | 1.24 (1.02-1.51) | 1.20 (0.71-2.00) |
| Mono | Reference | | | | | | |
| **UST vs Mono** |  |  |  |  |  |  |  |
| UST | 0.72 (0.58-0.88) | 1.87 (1.00-3.47) | 0.60 (0.41-0.88) | 0.63 (0.43-0.91) | 1.00 (0.42-2.40) | 0.81 (0.66-0.99) | 1.63 (0.74-3.56) |
| Mono | Reference | | | | | | |
| **UST vs Combo** |  |  |  |  |  |  |  |
| UST | 0.72 (0.59-0.89) | 1.19 (0.78-1.82) | 0.53 (0.33-0.84) | 0.44 (0.32-0.62) | 1.00 (0.42-2.40) | 0.63 (0.47-0.85) | 0.66 (0.35-1.25) |
| Combo | Reference | | | | | | |
| **VDZ vs Mono** |  |  |  |  |  |  |  |
| VDZ | 1.16 (1.43-0.95) | 1.08 (0.50-2.36) | 1.38 (0.73-2.60) | 0.79 (0.55-1.13) | 1.00 (0.42-2.40) | 1.07 (0.77-1.48) | 1.14 (0.58-2.21) |
| Mono | Reference | | | | | | |
| **VDZ vs Combo** | 1.21 (0.98-1.49) | 0.55 (0.33-0.92) | 1.21 (0.75-1.95) | 0.64 (0.48-0.85) | 1.00 (0.42-2.40) | 0.89 (0.69-1.16) | 0.78 (0.45-1.38) |
| VDZ |  |  |  |  | 1.00 (0.42-2.40) |  |  |
| Combo | Reference | | | | | | |
| **UST vs VDZ** | 0.68 (0.53-0.86) | 1.39 (0.96-2.02) | 0.50 (0.31-0.81) | 0.75 (0.56-0.99) | 1.00 (0.42-2.40) | 0.71 (0.56-0.91) | 0.87 (0.52-1.47) |
| UST |  |  |  |  |  |  |  |
| VDZ | Reference | | | | | | |

CI, confidence interval; Combo, TNFi combination therapy; IBD, inflammatory bowel diseases; Mono, TNFi monotherapy; RR, risk ratio; UST, ustekinumab; VDZ, vedolizumab
